# Development and Effects of a Metaverse-Based Generative AI Nursing Informatics Competency Education Program for Clinical Nurses

**DOI:** 10.3390/nursrep16090334

**Published:** 2026-09-11

**Authors:** Sang Un Park, So Young Choi

**Affiliations:** 1 Department of Nursing, Gyeongsang National University Hospital, 79 Gangnam-ro, Jinju-si 52727, Gyeongsangnam-do, Republic of Korea; tkddjs3@naver.com; 2 College of Nursing, Gyeongsang National University, 33 Dongjin-ro, Jinju-si 52725, Gyeongsangnam-do, Republic of Korea

**Keywords:** artificial intelligence, nursing informatics, clinical competence

## Abstract

**Background/Objectives**: The integration of generative artificial intelligence (AI) into healthcare has expanded the competencies required of clinical nurses. However, existing nursing informatics education programs do not adequately prepare nurses to use generative AI effectively and ethically in clinical practice. This study aimed to develop and evaluate a metaverse-based generative AI nursing informatics competency education program for clinical nurses. **Methods**: A randomized controlled pretest–posttest design was employed. Forty-eight clinical nurses were randomly assigned to an intervention group (*n* = 24) or a control group (*n* = 24), and 46 participants completed the study. The six-week education program was developed using the Analysis, Design, Development, Implementation, and Evaluation (ADDIE) instructional design model and Bandura’s self-efficacy theory. It was delivered through a metaverse platform using a flipped learning approach. Outcomes were measured at baseline, immediately after the intervention, and four weeks after the intervention. Data were analyzed using repeated-measures analysis of variance (ANOVA). **Results**: Compared with the control group, the intervention group demonstrated a significantly greater improvement in nursing informatics competency, the pre-specified primary outcome (*p* < 0.01), sustained at the four-week follow-up. Greater improvements were also observed for the secondary outcomes—general self-efficacy, ethical awareness of AI use, and evidence-based practice (all *p* < 0.01)—though these findings should be interpreted as hypothesis-generating given the absence of multiplicity adjustment. **Conclusions**: The metaverse-based generative AI education program improved self-reported nursing informatics competency and other key competencies among clinical nurses. **Trial Registration:** Clinical Research Information Service (CRIS) KCT0011821 registered on 8 April 2026.

## 1. Introduction

Recent advances in generative artificial intelligence (AI) are rapidly transforming healthcare delivery, education, and clinical decision-making processes. Powered by large language models, generative AI can generate, summarize, and synthesize information from diverse sources in response to users’ prompts, enabling healthcare professionals to efficiently retrieve evidence, support clinical reasoning, and develop patient education materials [1,2,3]. Consequently, generative AI is being increasingly integrated into healthcare systems as a clinical decision-support tool rather than merely an information retrieval technology [4,5]. However, because AI-generated outputs may contain inaccurate, incomplete, or fabricated information, healthcare professionals must possess the competency to critically evaluate AI-generated information and apply it appropriately in clinical practice [5,6,7].

Nurses are among the largest groups of healthcare professionals expected to utilize digital technologies in everyday clinical practice due to their roles as the primary point-of-care providers extensively engaged in the use of electronic health records, clinical decision-support systems, and AI-enabled tools. Accordingly, nursing informatics competency has become essential for delivering safe, evidence-based, and patient-centered care [8,9]. Nursing informatics competency has traditionally been defined as the ability to integrate computer technology, information management, and information systems into nursing practice [10]. More recently, the widespread adoption of generative AI has expanded this concept beyond conventional information retrieval to include the ability to effectively use AI technologies, critically appraise AI-generated information, and integrate AI-supported evidence into clinical decision-making [3,5,11]. Despite this growing demand, previous studies have reported that clinical nurses demonstrate only moderate levels of nursing informatics competency, indicating a substantial gap between the competencies required in rapidly evolving digital healthcare environments and those currently possessed by practicing nurses [12,13,14,15].

Existing nursing informatics education programs have primarily focused on conventional information technology skills, such as literature searching, electronic health record use, and information management [16,17,18]. Although these competencies remain important, they are insufficient for preparing nurses to safely and effectively utilize generative AI in clinical settings. Contemporary nursing practice requires nurses not only to operate AI technologies but also to critically evaluate AI-generated information, identify potential bias or misinformation, understand ethical issues related to AI use, and integrate AI-assisted evidence into evidence-based practice [5,6,19]. Furthermore, responsible AI use requires an understanding of privacy, algorithmic bias, transparency, explainability, accountability, and patient safety [7,20,21]. Therefore, educational interventions that simultaneously strengthen nursing informatics competency, AI ethical awareness, and evidence-based practice are urgently needed to address this gap [22,23,24].

To enhance learning effectiveness, educational programs should be grounded in a robust theoretical framework. Bandura’s self-efficacy theory proposes that individuals’ beliefs in their capability to perform specific tasks influence learning, motivation, and behavioral performance [25,26]. Self-efficacy can be strengthened through mastery experiences, vicarious experiences, verbal persuasion, and the regulation of emotional arousal [26]. Previous studies have consistently demonstrated that educational interventions incorporating self-efficacy enhancement strategies improve learners’ confidence and competence in applying newly acquired knowledge and skills [27,28]. Because nursing informatics competency requires repeated practice and confidence in applying digital technologies to authentic clinical situations, Bandura’s theory provides a suitable conceptual framework for designing educational interventions targeting clinical nurses and supports the inclusion of experiential and interactive learning strategies [18,29].

In addition to educational content, instructional methods are key determinants of educational effectiveness. Clinical nurses frequently encounter barriers to continuing education because of rotating shift schedules and heavy workloads [30,31]. Consequently, educational approaches that provide flexibility while maintaining high levels of learner engagement are required. Metaverse-based learning environments offer immersive and interactive learning experiences that enable learners to practice repeatedly in authentic clinical contexts without temporal or geographical constraints [32,33]. Previous studies have demonstrated that metaverse-based education improves knowledge acquisition, learner engagement, self-efficacy, and clinical performance among nursing students [32,34]. However, evidence regarding the effectiveness of metaverse-based education for clinical nurses, particularly when integrated with generative AI and evidence-based practice, remains limited [11,35].

Therefore, this study aimed to develop and evaluate a metaverse-based generative AI nursing informatics competency education program for clinical nurses based on the Analysis, Design, Development, Implementation, and Evaluation (ADDIE) instructional design model and Bandura’s self-efficacy theory. The program’s effectiveness was evaluated using a randomized controlled trial by examining its effects on nursing informatics self-efficacy, nurses’ ethical awareness of AI use, nursing informatics competency, and evidence-based practice.

## 2. Materials and Methods

### 2.1. Study Design

**Figure 1:**
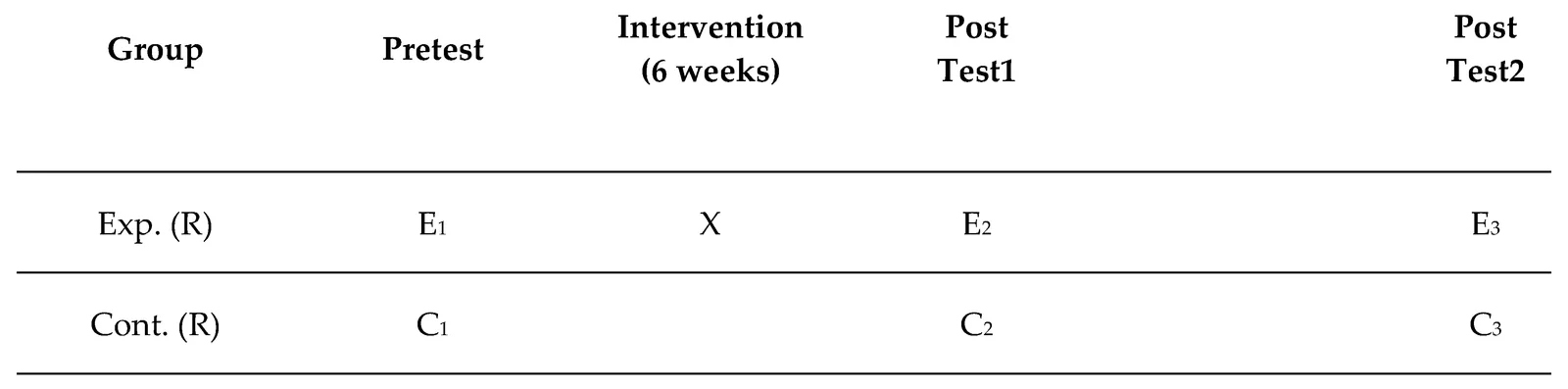
Research Design. R: randomization; Exp.: experimental group; Cont.: control group; X: metaverse-based generative AI nursing informatics competency education program; E_1_, C_1_: general characteristics, nursing informatics self-efficacy, nurses’ ethical awareness of AI use, nursing informatics competency, and evidence-based practice; E_2_, C_2_, E_3_, C_3_: nursing informatics competency self-efficacy, nurses’ ethical awareness of AI use, nursing informatics competency, and evidence-based practice.

This study employed a randomized controlled pretest–posttest design to develop and evaluate the effectiveness of a metaverse-based generative AI nursing informatics competency education program for clinical nurses. The intervention was developed according to the ADDIE instructional design model and evaluated using a randomized controlled trial. The program’s effectiveness was assessed by measuring nursing informatics self-efficacy, nurses’ ethical awareness of AI use, nursing informatics competency, and evidence-based practice at baseline, immediately after the intervention, and four weeks after the intervention. The study was designed and reported in accordance with the CONSORT 2025 statement [36]. The overall study design is presented in Figure 1.

### 2.2. Participants

Participants were registered nurses providing direct patient care services in general or tertiary hospitals in South Korea. Nurses serving in managerial positions (head nurse or above) or those not directly involved in patient care were excluded.

Participants were recruited through convenience sampling via an online nursing community between 11 and 18 February 2026. Convenience sampling was used because of feasibility constraints and the need to recruit clinically active nurses within a limited timeframe. Nurses who voluntarily agreed to participate and met the eligibility criteria were enrolled in this study.

Nursing informatics competency (NIC) was designated as the primary outcome of this study, consistent with the study’s primary aim. General self-efficacy, nurses’ ethical awareness of AI use, and evidence-based practice were treated as secondary outcomes.

The required sample size was calculated using G*Power version 3.1.9.7 [37] for repeated measures (analysis of variance [ANOVA]). Based on a previous nursing informatics competency education study [16], the effect size was set at f = 0.20, with a significance level of 0.05, statistical power of 0.80, three repeated measurements, a correlation coefficient of 0.50, and two study groups. The required minimum sample size was 21 participants per group. Considering a potential attrition rate of 10%, 48 participants were recruited.

Following completion of the baseline assessment, participants were randomly assigned to either the intervention or control group using the Research Randomizer computer-generated randomization program. The allocation sequence was concealed until completion of the baseline assessment and group assignment.

Data analysis was conducted using de-identified, coded data. The primary statistical analysis was performed by one of the authors, who was involved in the conduct of the study. To provide an independent check, the analysis was subsequently repeated by an external biostatistician who was not involved in intervention delivery or any other aspect of study conduct and is not a co-author of this manuscript.

**Figure 2:**
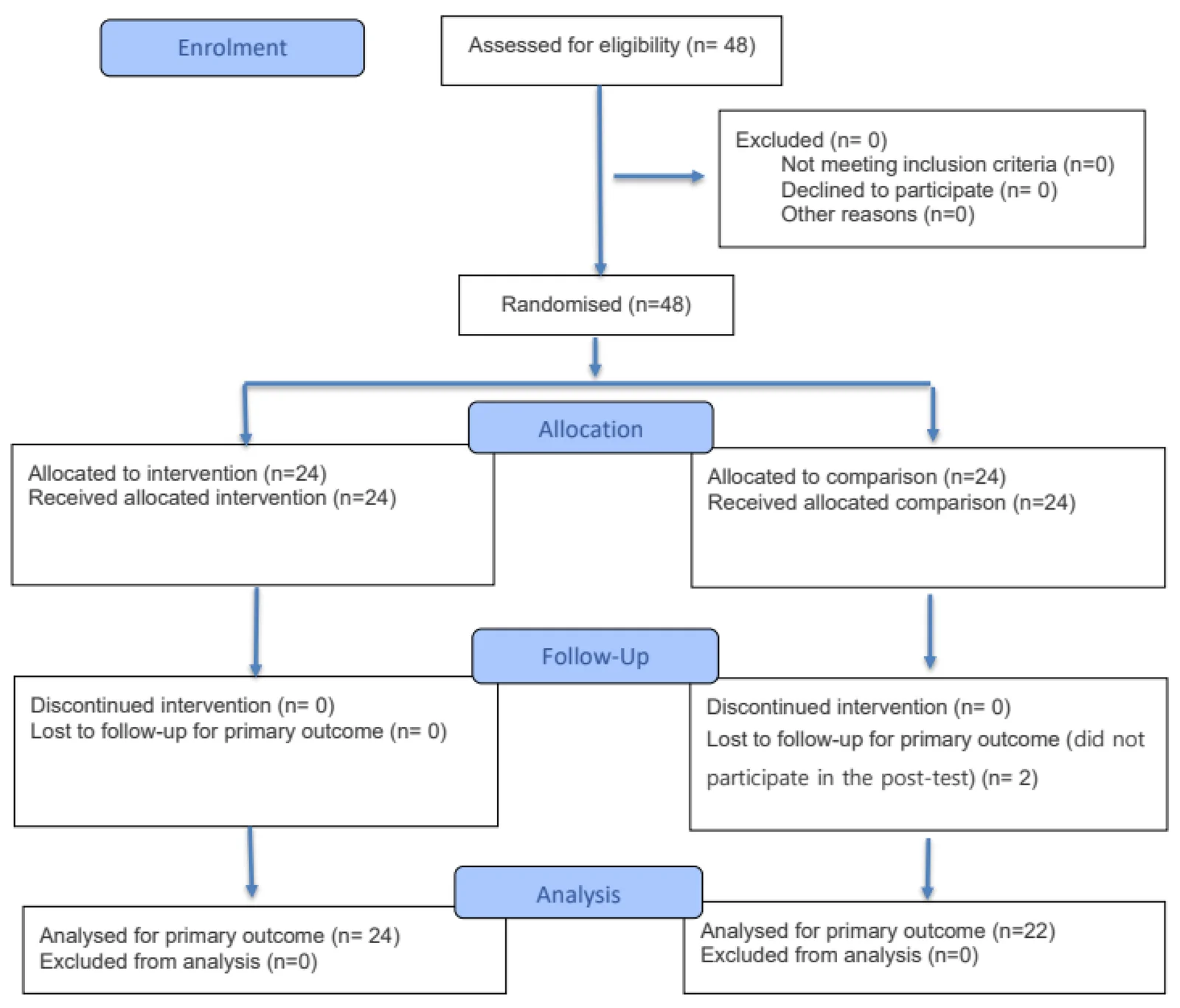
CONSORT 2025 flow diagram of participant recruitment and follow-up.

During the study, two participants in the control group withdrew due to an incomplete post-intervention assessment. Therefore, data from 46 participants (24 intervention; 22 control) were included in the final analysis. The participant flow is presented in Figure 2 in accordance with the CONSORT 2025 statement [36].

### 2.3. Outcome Measures

#### 2.3.1. General Self-Efficacy

General Self-Efficacy was measured using the Korean version of the General Self-Efficacy Scale (GSE) originally developed by Schwarzer and Jerusalem [38] and translated and validated by Ko and Kim [39]. The scale consists of 10 items rated on a 4-point Likert scale, ranging from 1 (“strongly disagree”) to 4 (“strongly agree”), with higher scores indicating greater general self-efficacy. Cronbach’s α ranged from 0.76 to 0.90 in the original instrument [38] and was 0.92 in the Korean version [39]; in this study, it was 0.87. At the time this study was designed, no validated instrument specifically measuring nursing informatics self-efficacy was available in the literature. The General Self-Efficacy Scale was therefore selected to assess participants’ general self-efficacy, which may be relevant to their confidence in learning and performing novel tasks, based on Bandura’s [25] self-efficacy theory.

Before responding to the GSE items, participants were instructed to consider their perceived ability to perform nursing-related tasks involving nursing information and data when rating each item. Although the original wording of the GSE items was not altered, this instructional framing provided a nursing informatics-related context for participants when responding to the items.

#### 2.3.2. Nurses’ Ethical Awareness of AI Use

Nurses’ ethical awareness regarding AI use was measured using the Nurses’ AI Ethical Awareness Scale developed by El-Sayed et al. [21]. Permission to use the instrument was obtained from the corresponding author. The instrument was translated into Korean following the World Health Organization (WHO) forward–backward translation guidelines. The translated version was reviewed by a bilingual nursing professor and two clinical nursing experts to ensure conceptual equivalence. Content validity was subsequently evaluated by a panel of four experts, comprising two nursing professors and two clinical nurses, yielding an item-level content validity index (I-CVI) of 0.75–1.00 and a scale-level content validity index (S-CVI/Average) of 0.96 before administration.

The instrument consists of 21 items across six domains: privacy, transparency, fairness, accountability, safety, and sustainability. Responses are rated on a 5-point Likert scale, ranging from 1 (“strongly disagree”) to 5 (“strongly agree”), with higher scores indicating greater ethical awareness regarding AI use. Cronbach’s α was 0.90 in the original instrument [21] and 0.86 in the present study.

#### 2.3.3. Nursing Informatics Competency

Nursing informatics competency was measured using the Korean Nursing Informatics Competency Assessment Scale (K-NICAS) developed by Jang [14] for clinical nurses.

The instrument consists of 20 items covering three domains: computer technology, information management, and information and communication technology. Each item is rated on a 4-point Likert scale, ranging from 1 (“strongly disagree”) to 4 (“strongly agree”), with higher scores indicating greater nursing informatics competency. Cronbach’s α was 0.91 in the original instrument [14] and 0.95 in the present study.

#### 2.3.4. Evidence-Based Practice

Evidence-based practice was measured using the Korean version of the Evidence-Based Practice Questionnaire (EBPQ) originally developed by Upton and Upton [40] and translated and validated for Korean nurses by Lim et al. [41].

The questionnaire consists of 24 items assessing practice, attitudes, and knowledge/skills related to evidence-based practice. Responses are rated on a 7-point Likert scale, with higher scores indicating greater evidence-based practice competency. Cronbach’s α was 0.87 in the original instrument [41], 0.95 in Lim et al.’s study [41], and 0.75 in the present study.

### 2.4. Development of the Metaverse-Based Generative AI Nursing Informatics Competency Education Program

The education program was developed according to the ADDIE instructional design model, comprising five sequential phases: analysis, design, development, implementation, and evaluation.

#### 2.4.1. Analysis

The analysis phase included a literature review, learner needs assessment, and learning environment analysis. Educational needs were identified through interviews with experienced clinical nurses and by reviewing nursing informatics competency frameworks, including QSEN, TIGER, and CASN. Based on the findings, three core educational domains—information management, information and communication technology, and information ethics—were derived.

#### 2.4.2. Design

Learning objectives, educational content, and instructional strategies were developed based on the needs assessment and Bandura’s self-efficacy theory. The program incorporated mastery experiences, vicarious learning, verbal persuasion, and emotional regulation to enhance nursing informatics self-efficacy.

Given the characteristics of shift-working nurses, the intervention adopted a flipped learning approach delivered through the ZEP metaverse platform.

#### 2.4.3. Development

Educational materials included pre-recorded video lectures, O/X quizzes, clinical scenarios, Padlet-based collaborative learning activities, and practical exercises using generative AI. Participants were encouraged to use generative AI platforms such as ChatGPT (GPT-5.2, OpenAI) and Gemini (Gemini 3 Pro, Google) to formulate prompts, search for evidence, critically appraise AI-generated responses, and solve authentic clinical problems.

#### 2.4.4. Implementation

The finalized program consisted of six weekly sessions, each lasting 80 min, including 20 min of asynchronous pre-class learning activities and 60 min of synchronous metaverse-based learning. Detailed session contents are presented in Table 1.

The session order was designed to progress from foundational concepts to applied use of generative AI. Session 1 introduced the concept and components of nursing informatics competency using three clinical scenarios (unfamiliar equipment after a ward rotation, an EMR-related case, and a PCA dose-adjustment case) to help learners identify competency gaps. Session 2 introduced ChatGPT as a clinical support tool and basic prompt engineering, using two scenarios (a COPD discharge-education case and a post-diagnosis case involving NGS testing and non-covered services) for prompt practice and reliability evaluation. Sessions 3–5 followed the evidence-based practice process using a common case—extending the heparin flush interval for a chemoport in a cancer patient—with Session 3 focused on PICO formulation, Session 4 on evidence searching, and Session 5 on quality appraisal using the SIGN checklist, each comparing learners’ own work with generative AI-assisted output. Session 6 concluded with a critical review of information-ethics issues in generative AI use. This design was intended to build practical AI-assisted competency, strengthen motivation through peer and instructor feedback, and foster critical, ethical evaluation of AI-generated information in clinical practice.

#### 2.4.5. Evaluation

The content validity of the program was evaluated by six experts in nursing education, nursing informatics, and clinical practice. After two rounds of expert review, the revised program demonstrated excellent content validity and was finalized for implementation.

### 2.5. Intervention

The intervention was delivered over six consecutive weeks using the ZEP metaverse platform. Before each session, participants completed approximately 20 min of self-directed pre-class learning through YouTube video lectures distributed via KakaoTalk.

During synchronous sessions, participants engaged in case-based discussions, collaborative learning using Padlet, and practical activities involving prompt engineering, AI-assisted evidence searching, critical appraisal of research evidence, and ethical decision-making. The educational activities emphasized repeated application of generative AI within authentic clinical scenarios.

### 2.6. Data Collection

Data were collected between 19 February and 30 April 2026, using structured online questionnaires administered through Google Forms.

Outcome variables were assessed at baseline, immediately after the intervention, and four weeks following completion of the program. Participants in both groups completed the assessments at identical time points.

Participants allocated to the control group received no study-related educational intervention during the six-week study period. They continued their usual clinical duties and did not receive any program-related materials or alternative educational activities during this period. After completion of the program and data collection, the PowerPoint (PPT) lecture materials were provided to the control group as an ethical consideration. Following completion of the study, participants in the control group received access to the same educational materials to ensure ethical fairness.

### 2.7. Statistical Analysis

Data were analyzed using IBM SPSS Statistics version 27.0.

Descriptive statistics were used to summarize participant characteristics and study variables. Normality assumptions were examined by conducting the Shapiro–Wilk test, and baseline homogeneity between groups was evaluated by conducting the chi-square test, Fisher’s exact test, the independent t-test, or the Mann–Whitney U test, as appropriate.

A complete-case analysis was conducted. The two participants in the control group who withdrew did not complete the outcome assessments at the second (immediately post-intervention) and third (4-week follow-up) time points; therefore, no outcome data were available for these participants beyond baseline, and they were excluded from the final analysis. An intention-to-treat sensitivity analysis was not performed given the relatively small sample size, which limits the reliability of imputation-based methods.

Intervention effects were analyzed using repeated-measures analysis of variance (RM-ANOVA) to examine group, time, and group-by-time interaction effects. Prior to conducting the RM-ANOVA, Mauchly’s test of sphericity was performed to assess whether the sphericity assumption was met. When the assumption of sphericity was violated, the Greenhouse–Geisser correction was applied. Effect sizes were reported using partial eta squared (ηp^2^) and interpreted according to Cohen’s criteria [42], whereby ηp^2^ values below 0.01 were considered a very small effect, 0.01 to below 0.06 a small effect, 0.06 to below 0.14 a medium effect, and 0.14 or greater a large effect. Statistical significance was established at *p* < 0.05. As nursing informatics competency was designated as the primary outcome, no adjustment for multiplicity across the four outcome variables was applied; findings for the secondary outcomes should therefore be interpreted as hypothesis-generating rather than confirmatory.

### 2.8. Ethical Considerations

This study was approved by the Institutional Review Board of G University (Approval No. GIRB-B25-NW-0092). **Trial Registration:** This trial was retrospectively registered with the Clinical Research Information Service (CRiS), Republic of Korea (Registration No. KCT0011821; registered on 8 April 2026). The trial was retrospectively registered due to administrative delays in completing the registration procedures; the primary and secondary outcomes, target sample size, and statistical analysis plan were specified in the IRB-approved protocol (approved on 7 February 2026, four days prior to the enrollment of the first participant on 11 February 2026) prior to the start of recruitment and were not modified after recruitment began or before outcome data were analyzed.

Electronic informed consent was obtained from all participants before data collection. Participants were informed of the study purpose, procedures, confidentiality, voluntary participation, and their right to withdraw without penalty. All collected data were anonymized and stored securely. After study completion, participants in the control group received the educational materials used in the intervention.

## 3. Results

### 3.1. Participant Characteristics

A total of 48 clinical nurses were enrolled in the study and randomly assigned to either the intervention (*n* = 24) or control (*n* = 24) group. During the study period, two participants in the control group withdrew because they did not complete the post-intervention assessment. Consequently, data from 46 participants (24 in the intervention group and 22 in the control group) were included in the final analysis (Figure 2).

Most participants were female (91.3%), aged between 20 and 39 years, held a bachelor’s degree (93.5%), and were employed as staff nurses (93.5%). Approximately two-thirds of the participants had between one and five years of clinical experience, and most participants had previously received nursing informatics education, primarily electronic medical record (EMR) training.

No statistically significant differences were observed between the intervention and control groups with respect to age, sex, educational level, clinical experience, job position, or previous nursing informatics education (all *p* > 0.05), indicating baseline equivalence between the two groups (Table 2).

### 3.2. Baseline Homogeneity of Outcome Variables

The normality assumptions for all outcome variables were assessed by conducting the Shapiro–Wilk test. All variables were normally distributed in both groups (all *p* > 0.05).

Baseline homogeneity of the outcome variables was subsequently evaluated. No statistically significant differences were identified between the intervention and control groups in nursing informatics self-efficacy, nurses’ ethical awareness of AI use, nursing informatics competency, or evidence-based practice prior to the intervention (all *p* > 0.05), confirming baseline equivalence between the groups (Table 3).

### 3.3. Effects of the Intervention

#### 3.3.1. General Self-Efficacy

In this exploratory analysis of a secondary outcome, general self-efficacy increased in the intervention group following completion of the six-week education program, with the improvement sustained at the four-week follow-up; by contrast, no comparable improvement was observed in the control group (Table 4).

Repeated-measures ANOVA demonstrated significant main effects of group (*F* = 63.06, *p* < 0.001) and time (*F* = 8.58, *p* < 0.001). Importantly, a significant group × time interaction was identified (*F* = 30.96, *p* < 0.001, ηp^2^ = 0.41), suggesting that the intervention may have led to greater improvements in general self-efficacy than the control condition. According to Cohen’s criteria [42], the observed interaction represented a large effect size; however, as this is a secondary outcome not adjusted for multiplicity, this finding should be considered hypothesis-generating.

#### 3.3.2. Nurses’ Ethical Awareness of AI Use

In this exploratory analysis of a secondary outcome, the intervention group showed a greater improvement in AI-related ethical awareness than the control group (Table 5).

Repeated-measures ANOVA revealed a significant main effect of group (*F* = 20.37, *p* < 0.001), whereas the main effect of time was not statistically significant (*F* = 0.96, *p* = 0.376). However, a significant group × time interaction was observed (*F* = 7.10, *p* = 0.003, ηp^2^ = 0.14), suggesting that ethical awareness may have improved over time only in the intervention group. The interaction effect was considered large; as a secondary outcome, however, this finding should be interpreted as hypothesis-generating rather than confirmatory.

#### 3.3.3. Nursing Informatics Competency

Nursing informatics competency significantly improved in the intervention group after completion of the program and remained above baseline at the four-week follow-up, whereas competency scores decreased slightly in the control group (Table 6).

Repeated-measures ANOVA demonstrated significant main effects of group (*F* = 47.10, *p* < 0.001) and time (*F* = 8.66, *p* = 0.001). A significant group × time interaction was also identified (*F* = 35.74, *p* < 0.001, ηp^2^ = 0.45), indicating greater improvements in the intervention group.

#### 3.3.4. Evidence-Based Practice

In this exploratory analysis of a secondary outcome, evidence-based practice scores increased in the intervention group following completion of the education program and remained improved four weeks later; by contrast, scores in the control group showed little change over time (Table 7).

Repeated-measures ANOVA revealed significant main effects of group (*F* = 92.56, *p* < 0.001) and time (*F* = 24.40, *p* < 0.001). A significant group × time interaction was also observed (*F* = 49.02, *p* < 0.001, ηp^2^ = 0.53), suggesting that the intervention may have substantially improved evidence-based practice compared with the control condition; as a secondary outcome not adjusted for multiplicity, this finding should be regarded as hypothesis-generating. Among the exploratory outcomes, evidence-based practice demonstrated the largest observed effect.

### 3.4. Adverse Events

No adverse events or unintended effects related to the metaverse-based generative AI Nursing informatics competency education program were reported during the study period.

## 4. Discussion

### 4.1. Principal Findings

This randomized controlled trial developed and evaluated a metaverse-based generative AI nursing informatics competency education program for clinical nurses based on the ADDIE instructional design model and Bandura’s self-efficacy theory. Overall, participants in the intervention group demonstrated a significantly greater improvement in nursing informatics competency, the pre-specified primary outcome, than those in the control group, with greater improvements also observed for the secondary, hypothesis-generating outcomes of general self-efficacy, ethical awareness of AI use, and evidence-based practice. Furthermore, these improvements were sustained at the four-week follow-up, suggesting that the educational effects extended beyond the immediate post-intervention period.

These findings suggest that integrating generative AI, metaverse-based learning, evidence-based practice, and self-efficacy enhancement strategies provides an effective educational approach for strengthening competencies required in contemporary digital healthcare environments [3,5,18,22,32,35].

### 4.2. Interpretation of the Findings

#### 4.2.1. General Self-Efficacy

As a secondary, hypothesis-generating finding, the intervention was associated with improved general self-efficacy, with the effect maintained four weeks after program completion. This finding is consistent with previous studies reporting that educational interventions based on Bandura’s self-efficacy theory effectively enhance learners’ confidence in applying newly acquired knowledge and skills in clinical practice [25,26,27,28,29].

A notable strength of the present intervention is that Bandura’s four sources of self-efficacy were intentionally incorporated into the instructional design rather than being applied solely as a theoretical framework. Mastery experiences were promoted through repeated practice using authentic clinical cases and generative AI tools. Vicarious experiences were facilitated through collaborative learning and peer presentations using the metaverse platform. Verbal persuasion was provided through continuous instructor feedback, while brief breathing exercises before each session supported emotional regulation. These complementary strategies may explain the sustained improvement in self-efficacy observed at the four-week follow-up [25,26,27,28,29].

Unlike conventional lecture-based nursing informatics education, participants repeatedly practiced prompt engineering, AI-assisted evidence searching, and critical evaluation of AI-generated information within realistic clinical scenarios. Such repeated application is likely to have strengthened learners’ confidence in using digital technologies in clinical practice [17,18,29].

#### 4.2.2. Nurses’ Ethical Awareness of AI Use

Another notable, though hypothesis-generating, finding was the improvement in nurses’ ethical awareness of AI use. As generative AI becomes increasingly integrated into healthcare, ethical competence has become an essential component of nursing informatics competency [5,6,7,21].

The improvement observed in this study may be attributed to the integration of authentic ethical case discussions with practical AI applications. Rather than focusing solely on ethical principles, participants analyzed realistic clinical scenarios involving privacy, algorithmic bias, accountability, transparency, and reliability of AI-generated information. This experiential approach encouraged participants to critically evaluate ethical dilemmas encountered during AI-supported clinical decision-making [20,21,22,23,24].

These findings support previous studies suggesting that ethics education is most effective when ethical concepts are learned through authentic clinical situations rather than through didactic instruction alone [21,22,23,24]. Accordingly, future AI education programs for nurses should integrate practical ethical reasoning into clinical simulations rather than present ethics as an isolated topic.

#### 4.2.3. Nursing Informatics Competency

Participants who completed the intervention demonstrated significant improvements in nursing informatics competency compared with the control group. This finding extends previous nursing informatics education studies by demonstrating that competency development can be enhanced through the integration of generative AI, metaverse-based experiential learning, and evidence-based practice [11,16,17,18].

Previous educational programs have primarily focused on computer literacy or electronic health record use. By contrast, the present intervention emphasized higher-order competencies, including prompt engineering, critical appraisal of AI-generated information, evidence searching, and clinical decision-making. Consequently, participants were encouraged not only to operate digital technologies but also to critically evaluate and appropriately apply AI-generated information in clinical practice [3,5,11,19].

These findings suggest that nursing informatics competency should be viewed as a multidimensional competency encompassing technological proficiency, critical thinking, ethical decision-making, and evidence-based clinical reasoning rather than simple information technology skills [9,10,11,12,13,14,15,16,17,18].

#### 4.2.4. Evidence-Based Practice

Among the secondary, hypothesis-generating outcomes, evidence-based practice demonstrated the largest observed effect. This is notable because evidence-based practice remains one of the most important competencies required for delivering safe, high-quality nursing care [24,41].

This improvement likely reflects the integration of generative AI into each step of the evidence-based practice process. Participants learned to formulate clinical questions using the PICO framework, search for relevant evidence using generative AI, critically evaluate research findings, and apply evidence to realistic clinical scenarios. These learning activities enabled participants to repeatedly experience the complete evidence-based practice process rather than learning individual concepts separately [3,19,24].

The findings suggest that generative AI has considerable potential as an educational tool for strengthening evidence-based practice when learners are simultaneously trained to critically evaluate AI-generated information. Rather than replacing nurses’ clinical reasoning, AI should be viewed as a decision-support technology requiring appropriate professional judgment [3,5,6,19].

### 4.3. Strengths and Implications

This study has several strengths. First, to our knowledge, this is among the first randomized controlled trials to develop and evaluate a metaverse-based generative AI nursing informatics competency education program specifically designed for clinical nurses. Second, the intervention integrated Bandura’s self-efficacy theory, evidence-based practice, AI ethics, and metaverse-based learning within a single educational framework, thereby addressing multiple competencies simultaneously [5,9,22,25,32]. Third, improvements were maintained at the four-week follow-up, suggesting sustained effects beyond the immediate post-intervention period.

The findings also have important implications for nursing education and clinical practice. The delivery of the intervention through an accessible metaverse platform, incorporating asynchronous and synchronous learning modalities, suggests its potential as a feasible and scalable continuing education model for shift-working clinical nurses [30,31,32,33,34,35]. Furthermore, integrating generative AI into nursing education may better prepare nurses for rapidly evolving digital healthcare environments while promoting safe, ethical, and evidence-based AI use in clinical practice [3,5,6,11].

### 4.4. Limitations

This study has several limitations.

First, participants were recruited using convenience sampling from among clinical nurses working in general and tertiary hospitals, which may limit the generalizability of the findings. The sample was also predominantly female (91.3%), aged 20–39 years, and held a bachelor’s degree (93.5%). These characteristics limit the generalizability of the findings to other groups, including male nurses, older nurses, nurses working in other types of institutions, and nurses in other countries.

Second, all outcome variables were measured using self-report questionnaires. Although validated instruments were used, self-reported data may be subject to response bias and reflect participants perceived rather than objectively assessed competencies. Therefore, the observed improvements should be interpreted as changes in perceived or self-reported nursing informatics competency and self-efficacy rather than objective competency.

Third, the follow-up period was limited to four weeks after intervention completion. Consequently, the long-term sustainability of the intervention effects could not be determined.

Fourth, the effect sizes observed in this study (partial ηp^2^ = 0.14–0.53) were larger than the effect size used in the a priori sample size calculation (f = 0.20). The education program was designed to incorporate all four sources of self-efficacy proposed by Bandura [25]—mastery experience, vicarious experience, verbal persuasion, and emotional and physiological states—which may partly account for the larger-than-expected effects. However, because all outcome variables were measured using self-report instruments, we cannot rule out the possibility that these effect sizes were inflated by response biases such as the Hawthorne effect (participants modifying their responses due to awareness of being observed) or social desirability bias (participants rating themselves more favorably following a novel, closely monitored intervention). This explanation cannot be fully verified with the present data, and future studies should incorporate objective or behavioral measures of competency, alongside methods to assess and control for social desirability, to corroborate the magnitude of the effects observed here. In addition, because the control group received no program-related educational materials or alternative activity during the six-week study period, this no-treatment design cannot fully distinguish the effects of the educational content itself from those attributable to the novelty of the metaverse-based, AI-assisted learning format. PowerPoint lecture materials were provided to the control group after completion of the program and data collection for ethical considerations. Future studies should consider an active comparison condition to help disentangle these influences.

Fifth, baseline familiarity with generative AI tools and metaverse platforms was not formally assessed; therefore, we cannot statistically confirm that the intervention and control groups were equivalent on these characteristics prior to the intervention. Future studies should assess and control for baseline familiarity with AI and metaverse technologies to more clearly isolate the effect of the educational content itself.

Sixth, nursing informatics self-efficacy was measured using a general self-efficacy scale rather than a domain-specific instrument, as no validated tool for this construct was available at the time of the study. Future research should develop and validate a nursing informatics-specific self-efficacy scale.

Finally, control-group scores declined between baseline and follow-up assessments for several self-reported outcome variables (general self-efficacy, nursing informatics competency, and evidence-based practice). The raw data entry and score calculations for the control group were re-verified across all three time points, and no coding or data-entry errors were identified.

This decline should not be interpreted as an actual loss of competence, but rather considered in light of the self-report nature of the instruments used in this study. Participants repeatedly exposed to the same items may have come to recognize the meaning and expected level of these competencies more concretely over time and consequently applied a stricter self-evaluation standard at follow-up. This pattern is therefore more plausibly attributable to a shift in participants’ internal standard of self-assessment upon repeated measurement—commonly referred to as response shift—than to an actual decline in competence resulting from the absence of education [43,44].

A second possible contextual factor is the timing of data collection. Data were collected between February and April 2026. In South Korea, this period may coincide with periods of increased workload and organizational transition in clinical settings. However, because workload and related contextual factors were not directly measured in this study, we cannot determine whether, or to what extent, these factors contributed to the observed decline in the control group. Therefore, this possibility should be regarded as a plausible but unverified contextual consideration.

A third possible explanation is resentful demoralization, whereby control-group participants, aware that they had not been assigned to the metaverse-based intervention, may have experienced reduced motivation or more critical self-appraisal at follow-up. As with the contextual timing factor discussed above, this possibility was not directly assessed in this study, and its actual contribution to the observed decline cannot be confirmed. We acknowledge this as an additional plausible, though unverified, explanation alongside response shift and contextual timing factors.

A substantial portion of the observed group-by-time interaction may therefore reflect control-group deterioration—arising from response shift and, possibly, contextual factors—in addition to genuine improvement in the intervention group. Future studies should directly measure contextual workload factors and consider retrospective pretest (then-test) designs to assess response shift, alongside objective competency measures and an active comparison condition, to more clearly isolate true intervention effects.

In addition, although the study protocol—including the primary and secondary outcomes, sample size, and statistical analysis plan—was finalized and approved by the IRB on 7 February 2026, prior to the enrollment of the first participant, the trial was not registered with a public clinical trial registry until 8 April 2026, after data collection had already begun. This retrospective registration does not meet the prospective registration standard recommended by ICMJE and CONSORT guidelines, which is intended to prevent outcome switching and publication bias. While the IRB-approved protocol provides documentary evidence that the study design was fixed before recruitment, this does not fully substitute for the transparency afforded by prospective public registration, and we acknowledge this as a limitation of the study.

Finally, the PowerPoint materials provided to the control group after completion of data collection offered access to the core educational content but did not replicate the interactive, metaverse-based learning experience delivered to the intervention group. This content-only provision does not constitute an equivalent standard of education, and future studies should consider offering control-group participants fuller access to the interactive program components once data collection is complete, where feasible.

## 5. Conclusions

This study developed and evaluated a metaverse-based generative AI nursing informatics competency education program for clinical nurses using the ADDIE instructional design model and Bandura’s self-efficacy theory. The six-week intervention improved perceived nursing informatics competency, the pre-specified primary outcome, in the intervention group compared with the control group. Improvements were also observed for the secondary outcomes of self-reported general self-efficacy, nurses’ ethical awareness of AI use, and evidence-based practice; however, as these were not adjusted for multiplicity, they should be regarded as hypothesis-generating findings requiring confirmation in future adequately powered studies.

The findings suggest that integrating generative AI, metaverse-based learning, flipped learning, and theory-driven instructional strategies may support the development of perceived digital competencies required in contemporary nursing practice. Combining AI-assisted evidence searching, authentic clinical scenarios, collaborative learning, and ethical decision-making may provide a useful educational model for preparing nurses to work in increasingly digital healthcare environments.

Future multicenter studies with larger and more diverse samples, longer follow-up periods, and objective and performance-based measures of competency are needed to determine whether the improvements observed in self-reported outcomes translate into actual clinical competency and behavioral change, and to establish the long-term effectiveness and broader applicability of this educational approach.

## Figures and Tables

**Table 1:** Metaverse-based generative AI nursing informatics competency education program.

Session	Main Theme	Contents and Activities	Self-Efficacy Strategy	Time (min)	Methods/ Materials	Expected Outcomes
1	Nursing Informatics Competency (NIC)	**Pre-class** OT; definition, necessity, and components of NIC Role of NIC in clinical decision-making; CASN NIC	-	20	PPT video lecture	GSE, NIC
		**Intro** Team introductions RBS ^†^;	PA	60		GSE
		**Activity** Small-group discussion on NIC challenges; -Identify Informatics Competency Gaps Through CASN-Based Clinical Case Analysis	ME, VE		Discussion/PowerPoint slides	GSE, NIC
		**Conclusion** Summary of Learning	VE			GSE
		Report Sharing and Feedback	VP			
		Next Session Briefing	-			
2	Generative AI as an Assistive Tool	**Pre-class** Review; generative AI as a support tool; role of prompts; prompt-engineering principles; usage considerations	-	20	PPT video lecture	GSE, NAIEA, NIC
		**Intro** Pre-learning O/X quiz RBS ^†^;	ME	60	O/X quiz	GSE, NAIEA, NIC
		**Activity** Individual Padlet activity: prompt creation/search/sharing for clinical scenarios; reliability evaluation and reflection	ME, VE, PA		Discussion/Padlet, ChatGPT	GSE, NAIEA, NIC, EBPC
		**Conclusion** Sharing of Outputs and Feedback	VE, VP			GSE, NAIEA, NIC
		Review of Key Learning Points	ME			
		Preview of the Next Session	-			
3	EBP Using Generative AI (PICO)	**Pre-class** Definition and importance of EBP; PICO concept and components; benefits/limits of AI for question refinement	-	20	PPT video lecture	GSE, NIC, EBP
		**Intro** Pre-learning O/X quiz RBS ^†^;	ME	60	O/X quiz	GSE, NIC, EBP
		**Activity** Individual Padlet activity: Development of Case-Based PICO Questions	ME		Discussion/Padlet, ChatGPT	GSE, NAIEA, NIC, EBP
		Development of AI-Assisted PICO Questions	ME			
		Comparative Analysis of Self-Generated and AI-Assisted PICO Questions	VE, ME			
		Discussion of Considerations for Generative AI Use	VP			
		**Conclusion** Sharing of Outputs and Feedback	VE, VP			GSE, NIC
		Review of Key Learning Points	ME			
		Preview of the Next Session	-			
4	EBP Using Generative AI (Evidence Search)	**Pre-class** PICO-based search-strategy development; major databases; AI-assisted keyword expansion and searching; considerations	-	20	PPT video lecture	GSE, NIC, EBP
		**Intro** Pre-learning O/X quiz, RBS ^†^;	ME	60	O/X quiz	GSE, NIC
		**Activity** Individual Padlet activity: AI-Assisted Evidence Search	VE		Discussion/Padlet, ChatGPT	GSE, NIC, EBP
		Evaluation of the Reliability of AI-Generated Evidence and Discussion of Precautions	VP, ME			
		Development of Personalized Prompts to Improve the Accuracy of AI-Generated Responses	ME			
		**Conclusion** Sharing of Outputs and Feedback	VE, VP			GSE, NIC
		Review of Key Learning Points	ME			
		Preview of the Next Session	-			
5	EBP Using Generative AI (Quality Appraisal)	**Pre-class** Definition/importance of critical appraisal; SIGN tools by study design; AI limitations (e.g., hallucinations)	-	20	PPT video lecture	GSE, NIC
		**Intro** Pre-learning O/X quiz, RBS ^†^;	ME	60	O/X quiz	GSE, NIC
		**Activity** Individual Padlet activity: Critical Appraisal of Retrieved Evidence Using the SIGN Checklist	ME		Discussion/Padlet, ChatGPT	GSE, NAIEA, NIC, EBP
		Comparison of Appraisal Results According to AI Input Conditions	VE, ME			
		Preparation of an Analytical Report on Appraisal Methods and Findings	ME			
		**Conclusion** Sharing of Outputs and Feedback	VE, VP			GSE, NIC
		Review of Key Learning Points	ME			
		Preview of the Next Session	-			
6	Information Ethics	**Pre-class** AI ethical issues; Korean Personal Information Protection Act and Bioethics and Safety Act; EU AI Act; medical ethics in the AI era; ethical principles for healthcare AI use	-	20	PPT video lecture	NAIEA
		**Intro** Pre-learning O/X quiz, RBS ^†^;	ME	60	O/X quiz	GSE, NAIEA, NIC
		**Activity** Individual Padlet activity: Discussion of Clinical Cases Based on Generative AI Ethical Principles	VP, VE		Discussion/Padlet, Google Forms questionnaire	GSE, NAIEA, NIC
		Preparation of a Discussion Report	ME			
		**Conclusion** Sharing of Outputs and Feedback	VE, VP			GSE, NIC
		Review of Key Learning Points	ME			
		Post-Program Survey	-			

Note. GSE = general self-efficacy; NIC = nursing informatics competency; EBP = evidence-based practice; NAIEA = nurses’ ethical awareness of AI use. Self-efficacy strategy: PA = physiological arousal; ME = mastery experience; VE = vicarious experience; VP = verbal persuasion. RBS ^†^ = relaxation breathing stretch, performed at the beginning of each activity phase.

**Table 2:** Homogeneity test of participants’ general characteristics.

Characteristics	Categories	Total (*n* = 46)	Exp. (*n* = 24)	Cont. (*n* = 22)	χ^2^/t	*p*
		*n* (%)	*n* (%)	*n* (%)		
Gender	Male	4 (8.7)	3 (12.5)	1 (4.5)		0.609 ^†^
	Female	42 (91.3)	21 (87.5)	21 (95.5)		
Age (years)	20–29	26 (56.5)	14 (58.3)	12 (54.5)	0.07	0.796
	30–39	20 (43.5)	10 (41.7)	10 (45.5)		
Education level	Bachelor’s degree	43 (93.5)	24 (100)	19 (86.4)		0.101 ^†^
	Master’s degree	3 (6.5)	0 (0.0)	3 (13.6)		
Job position	Staff nurse	43 (93.5)	24 (100)	19 (86.4)		0.101 ^†^
	Specialized nurse	3 (6.5)	0 (0.0)	3 (6.5)		
Clinical experience (years)	1–5	29 (63.0)	18 (75.0)	11 (50.0)	3.08	0.079
	>5	17 (37.0)	6 (25.0)	11 (50.0)		
Experience with nursing informatics education (including EMR education)	Yes	41 (89.1)	23 (95.8)	18 (91.8)		0.178 ^†^
	No	5 (10.9)	1 (4.2)	4 (18.2)		

Exp.: experimental group, Cont.: control group; EMR: electronic medical record; ^†^ Fisher’s exact test.

**Table 3:** Homogeneity test of pretest outcome variables between the experimental and control groups (*N* = 46).

Variables	Exp. (*n* = 24)	Cont. (*n* = 22)	t	*p*
	M ± SD	M ± SD		
Nursing informatics self-efficacy	3.43 ± 0.13	3.42 ± 0.14	0.26	0.797
Nurses’ ethical awareness of AI use	3.52 ± 0.10	3.46 ± 0.13	1.60	0.116
Nursing informatics competency	3.39 ± 0.15	3.33 ± 0.10	1.62	0.112
Evidence-based practice	6.04 ± 0.18	6.07 ± 0.17	−0.66	0.515

Exp.: experimental group, Cont.: control group.

**Table 4:** Differences in general self-efficacy between the experimental and control groups (*N* = 46).

Group	Time			Time Comparison		Source	*F* (*p*)	ηp^2^
	Pretest	Posttest1	Posttest2	Pre–Post1	Pre–Post2			
	M ± SD	M ± SD	M ± SD	t(*p*)	t(*p*)			
Exp. (*n* = 24)	3.43 ± 0.13	3.59 ± 0.11	3.63 ± 0.08	−4.66 (<0.001)	−6.10 (<0.001)	Group	63.06 (<0.001)	
Cont. (*n* = 22)	3.42 ± 0.15	2.84 ± 0.56	2.88 ± 0.48	4.53 (<0.001)	5.17 (<0.001)	Time	8.58 (<0.001)	
						Group × Time	30.96 (<0.001)	0.41

Exp.: experimental group, Cont.: control group; ηp^2^: partial eta squared.

**Table 5:** Differences in nurses’ ethical awareness of AI use between the experimental and control groups (*N* = 46).

Group	Time			Time Comparison		Source	*F*(*p*)	ηp^2^
	Pretest	Posttest1	Posttest2	Pre–Post1	Pre–Post2			
	M ± SD	M ± SD	M ± SD	t(*p*)	t(*p*)			
Exp. (*n* = 24)	3.52 ± 0.10	3.63 ± 0.07	3.67 ± 0.07	−4.82 (<0.001)	−5.48 (<0.001)	Group	20.37 (<0.001)	
Cont. (*n* = 22)	3.46 ± 0.13	3.31 ± 0.41	3.37 ± 0.34	1.61 (0.121)	1.40 (0.177)	Time	0.96 (0.376)	
						Group × Time	7.10 (0.003)	0.14

Exp.: experimental group, Cont.: control group; ηp^2^: partial eta squared.

**Table 6:** Difference in nursing informatics competency between the experimental and control groups.

Group	Time			Time Comparison		Source	*F* (*p*)	ηp^2^
	Pretest	Posttest1	Posttest2	Pre–Post1	Pre–Post2			
	M ± SD	M ± SD	M ± SD	t(*p*)	t(*p*)			
Exp. (*n* = 24)	3.39 ± 0.15	3.63 ± 0.11	3.53 ± 0.10	−6.13 (<0.001)	−3.67 (0.001)	Group	47.10 (<0.001)	
						Time	8.66 (0.001)	
Cont. (*n* = 22)	3.33 ± 0.10	2.64 ± 0.62	2.83 ± 0.61	5.50 (<0.001)	3.96 (0.001)	Group × Time	35.74 (<0.001)	0.45

Exp.: experimental group, Cont.: control group; ηp^2^: partial eta squared.

**Table 7:** Difference in evidence-based practice between the experimental and control groups (*N* = 46).

Group	Time			Time Comparison		Source	F (*p*)	ηp^2^
	Pretest	Posttest1	Posttest2	Pre–Post1	Pre–Post2			
	M ± SD	M ± SD	M ± SD	t(*p*)	t(*p*)			
Exp. (*n* = 24)	6.04 ± 0.18	6.20 ± 0.15	6.24 ± 0.22	−3.08 (0.005)	−3.40 (0.002)	Group	92.56 (<0.001)	
Cont. (*n* = 22)	6.06 ± 0.17	5.78 ± 0.34	5.07 ± 0.57	3.72 (0.001)	7.67 (<0.001)	Time	24.40 (<0.001)	
						Group ×Time	49.02 (<0.001)	0.53

Exp.: experimental group, Cont.: control group; ηp^2^: partial eta squared.

## Data Availability

The data presented in this study are available from the corresponding author upon reasonable request. The data are not publicly available because they contain information that could compromise the privacy of the research participants.

## Public Involvement Statement

No public was involved in any aspect of this research.

## Guidelines and Standards Statement

This manuscript was prepared in accordance with the CONSORT 2025 Statement (Consolidated Standards of Reporting Trials) for randomized controlled trials.

## Abbreviations

The following abbreviations are used in this manuscript:

ADDIE	Analysis, Design, Development, Implementation, Evaluation
AI	Artificial intelligence
AIEA	Artificial intelligence ethical awareness
ANOVA	Analysis of variance
CASN	Canadian Association of Schools of Nursing
CONSORT	Consolidated Standards of Reporting Trials
EBP	Evidence-based practice
EBPQ	Evidence-Based Practice Questionnaire
EMR	Electronic medical record
EU	European Union
Exp.	Experimental group
GSE	General Self-Efficacy Scale
K-NICAS	Korean Nursing Informatics Competency Assessment Scale
ME	Mastery experience
NAIEA	Nurses’ ethical awareness in the use of artificial intelligence
NIC	Nursing informatics competency
NIC-SE	Nursing informatics competency self-efficacy
O/X	True/False (Correct/Incorrect)
OT	Orientation
PA	Physiological arousal
PICO	Population, Intervention, Comparison, Outcome
QSEN	Quality and Safety Education for Nurses
RBS	Relaxation breathing stretch
RM-ANOVA	Repeated-measures analysis of variance
SPSS	Statistical Package for the Social Sciences
TIGER	Technology Informatics Guiding Education Reform
VE	Vicarious experience
VP	Verbal persuasion
WHO	World Health Organization
